# Muscle strength is longitudinally associated with mobility among older adults after acute hospitalization: The Hospital-ADL study

**DOI:** 10.1371/journal.pone.0219041

**Published:** 2019-07-05

**Authors:** Jesse J. Aarden, Marike van der Schaaf, Martin van der Esch, Lucienne A. Reichardt, Rosanne van Seben, Jos A. Bosch, Jos W. R. Twisk, Bianca M. Buurman, Raoul H. H. Engelbert, on behalf of the Hospital-ADL study group

**Affiliations:** 1 Amsterdam UMC, Academic Medical Center, University of Amsterdam, Department of Rehabilitation, Amsterdam Movement Sciences, Amsterdam, Netherlands; 2 Amsterdam Center for Innovative Health Practice (ACHIEVE), Faculty of Health, Amsterdam University of Applied Sciences, Amsterdam, Netherlands; 3 European School of Physiotherapy, Faculty of Health, Amsterdam University of Applied Sciences, Amsterdam, Netherlands; 4 Reade, Center for Rehabilitation and Rheumatology/Amsterdam Rehabilitation Research Center, Amsterdam, Netherlands; 5 Amsterdam UMC, Academic Medical Center, University of Amsterdam, Department of Internal Medicine, Section of Geriatric Medicine, Amsterdam, Netherlands; 6 Department of Clinical Psychology, University of Amsterdam, Amsterdam, Netherlands; 7 Amsterdam UMC, Vrije Universiteit Amsterdam, Department of Epidemiology and Biostatistics, Amsterdam, Netherlands; Medical University Graz, AUSTRIA

## Abstract

**Background:**

30 to 60% of the acute hospitalized older adults experience functional decline after hospitalization. The first signs of functional decline after discharge can often be observed in the inability to perform mobility tasks, such as raising from a chair or walking. Information how mobility develops over time is scarce. Insight in the course of mobility is needed to prevent and decrease mobility limitations.

**Objectives:**

The objectives of this study were to determine (i) the course of mobility of acute hospitalized older adults and (ii) the association between muscle strength and the course of mobility over time controlled for influencing factors.

**Methods:**

In a multicenter, prospective, observational cohort study, measurements were taken at admission, discharge, one- and three months post-discharge. Mobility was assessed by the De Morton Mobility Index (DEMMI) and muscle strength by the JAMAR. The longitudinal association between muscle strength and mobility was analysed with a Linear Mixed Model and controlled for potential confounders.

**Results:**

391 older adults were included in the analytic sample with a mean (SD) age of 79.6 (6.7) years. Mobility improved significantly from admission up to three months post-discharge but did not reach normative levels. Muscle strength was associated with the course of mobility (beta = 0.64; p<0.01), even after controlling for factors as age, cognitive impairment, fear of falling and depressive symptoms (beta = 0.35; p<0.01).

**Conclusion:**

Muscle strength is longitudinally associated with mobility. Interventions to improve mobility including muscle strength are warranted, in acute hospitalized older adults.

## Introduction

After acute hospitalization, 30 to 60% of older adults ≥65 years of age experience functional decline, resulting in limitations of activities of daily life, unplanned readmissions to hospital or even death [1–5]. The first signs of functional decline can often be observed in the inability to perform mobility tasks, such as raising from a chair or walking [6].

Recent studies [7,8] showed that mobility is impaired in most older adults at the time of acute hospital admission. Despite an improvement during and after hospitalization, mobility levels remain below reference levels up to one-month post-discharge [7–9]. While it has been suggested that after hospitalization, three months might be needed to regain mobility to the level before hospitalization [3], no information is available on the course of mobility over a longer time period as well as influencing factors, that might affect the course.

Muscle strength is considered as an essential prerequisite for mobility and muscle weakness and is associated with reduced mobility and functional decline [10,11]. The role of muscle strength in the development of mobility limitations is best explained through the concept of functional reserve capacity: individuals with relative higher muscle strength are relatively less affected in their mobility than older adults with low muscle strength [12]. Hence, it is conceivable that muscle strength plays an important role in reduced mobility and recovery, over the post-discharge course [13–15].

Besides muscle strength, factors such as age, cognitive impairment, depressive symptoms, fear of falling, fatigue and nutrition have been associated with reduced mobility and functional decline after acute hospitalization [16–20]. These factors may be barriers to regain mobility and may interact with muscle strength. A better understanding of the longitudinal association between muscle strength and the course of mobility over a longer time-period post-discharge and the influence of demographic- and psychosocial factors will help to understand the mechanisms of reduced mobility. This insight could help to develop tailored interventions to improve the level of mobility and daily functioning in acute hospitalized older adults.

Therefore, the aims of this longitudinal study were to determine: (i) the course of mobility from admission up to three months post-discharge, (ii) the association between muscle strength and the course of mobility and (iii) the role of demographic and psychosocial factors in this association up to three months post-discharge, in acute hospitalized older adults.

## Methods

### Design and setting

The Hospital-Associated Disability and impact on daily Life (Hospital-ADL) study, a multicenter observational prospective cohort study, was conducted by a multidisciplinary geriatric team. Participants were recruited from those who were admitted to the wards of Internal Medicine, Cardiology or Geriatrics at six participating hospitals in the Netherlands. The study was approved by the Institutional Review board of the Amsterdam UMC, Academic Medical Center (AMC) in The Netherlands (Protocol ID: AMC2015_150) and performed according to the Dutch Medical Research Involving Human Subjects Act and principles of the Declaration of Helsinki (1964). Local approval was additionally provided by all participating hospitals.

### Participants

Older adults aged ≥70 years who were acutely admitted for at least 48 hours were approached for participation. In addition, further inclusion criteria were applied: 1] approval of the attending Medical doctor; 2] Mini-Mental State Examination (MMSE) score ≥ 15; 3] sufficient Dutch language proficiency to complete questionnaires. Older adults were excluded if they 1] had a life expectancy of less than three months, as assessed by the attending Medical Doctor; 2] were disabled in all six basic ADL’s as determined by the Katz-ADL index.

### Data collection

LR and RS visited the participating wards and contacted all eligible patients within 48 hours after hospital admission. After informed consent was obtained, older adults were enrolled in the study. The geriatric team completed interviews and executed performance tests with participants at baseline (T_0_) (within 48 hours after admission), discharge (T_1_) and at one- (T_2_) and three months (T_3_) post-discharge (at participants home or residence). The researchers were trained to administer the study protocol in order to reduce variability. Data was collected between October 1, 2015 and June 1, 2017.

### Mobility

Mobility was assessed with the De Morton Mobility Index (DEMMI). The DEMMI is a unidimensional mobility measure for older adults making the transition from hospital to the community and based on Rasch analysis. The DEMMI consists of 15 items and a raw ordinal score is converted to an interval-level score out of 100. Higher scores indicate a better mobility performance. Older adults are considered as independent for daily living with a score of 74. Previous studies showed good reliability and validity in studies with older adults during and after hospitalization. The reported minimal clinical important difference was 10 points [9,21]. The DEMMI consists of the following items: perform a bridge, roll onto side, lie to sit, sit unsupported in chair, sit to stand from chair, sit to stand without using arms, stand unsupported, stand feet together, stand on toes, tandem stand, walking distance, walking assistance, pick up pen from floor, walk backwards, and jump. Participants were asked to perform these tasks and were scored according to the standardized protocol.

### Muscle strength

Muscle strength was measured using a Jamar handgrip strength dynamometer (Lafayette Instrument Company, USA). The handgrip strength was measured to provide an objective index of general upper body strength. Handgrip strength showed good to excellent test-retest reliability and interrater reliability and good validity among hospitalized older adults [22]. Normative values of older adults are available from Dodds et al. (2014) for gender related age groups [12]. We considered muscle strength lower than one standard deviation of the mean score as decreased muscle strength. Participants were measured in supine or sitting position and encouraged to show maximal isometric handgrip strength and performed the task thrice bilaterally. The highest score (in kilogram) of both hands was used for the analysis.

### Other variables

Confounding variables, possibly affecting the association of muscle strength with course of mobility, were assessed. Participants were assessed on 1) cognitive impairment with the Mini Mental State Examination (MMSE) [23]; 2) depressive symptoms with the Geriatric Depression Scale-15 (GDS-15) [24], 3) fatigue and fear of falling (FOF) using a 10-point numeric rating scale; 4) number and severity of comorbidities with the Charlson Comorbidity Index (CCI) [25]; 5) malnourishment with the Short Nutritional Assessment Questionnaire (SNAQ) [26]. In addition, mean age, length of stay, highest level of education, marital status, living arrangement, length of stay in hospital (LOS) and Body Mass Index (BMI) were collected [27].

### Statistical analysis

Baseline characteristics were calculated using descriptive statistics. Data was checked on normality by plotting histograms of the residuals. A Linear Mixed Model (LMM) was performed to analyse the course of mobility and the association between the course of mobility and muscle strength. In this procedure it is not essential to use multiple imputation of missing data before performing the LMM [28]. To evaluate the effect of potential confounders (gender, age, cognitive impairment, depressive symptoms, fear of falling and fatigue) on this association, variables were stepwise added to the model. For every potential confounder it was determined if the beta (*β*) in the association between muscle strength and mobility changed with more than 10%. A 10% change of the regression coefficient of the determinant in the crude model after adjustment for one factor was indicative for relevant confounding. Finally, confounding on the association of muscle strength with the course of mobility, was determined, based on a 10% change of the regression coefficient again. Prior to these analyses, interaction effects between muscle strength and time, gender and age in the association with the course of mobility were calculated to analyse whether stratification was needed.

To analyse if the associations between muscle strength and mobility was similar for older adults with decreased muscle strength, a sensitivity analysis was performed. All parameter estimates were expressed with a 95% confidence interval (95%CI), and results were considered significant if p<0.05. Analyses were conducted with the SPSS Statistics (version 24.0).

## Results

### Characteristics of the study sample

**Fig 1:**
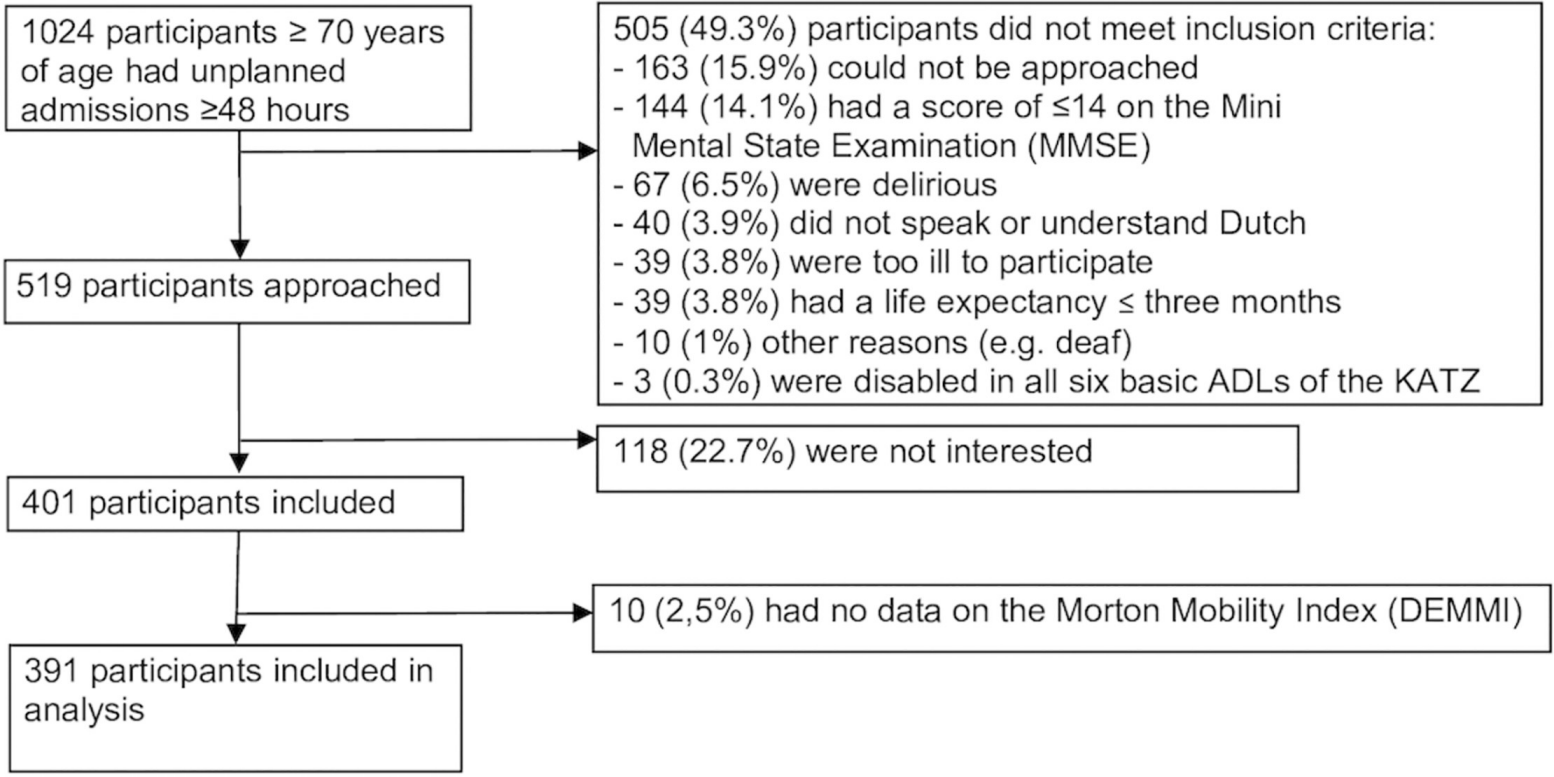
Inclusion of participants in the study (N = 391).

1024 acute hospitalized older adults were admitted to the participating hospital wards ≥48 hours. Of these unplanned admissions, 519 (50.7%) participants met the inclusion criteria and were approached, of whom 401 (77.3%) participants agreed to participate. Participants were excluded because they were not approachable (163 (15.9%)), a score ≤14 on the MMSE (144 (14.1%)), were delirious (67 (6.5%)), did not speak or understand Dutch (40 (3.9%)), were too ill to participate (39 (3.8%)), had a life expectancy of ≤3 months (39 (3.8%)) or other reasons (13 (1.3%)) (e.g. deaf, disabled in all six basic ADLs). Ten participants (2.5%) had no data for the DEMMI at any of the time points and were excluded from the sample. Finally, 391 older adults were included in the statistical analysis (Fig 1).

Data of the DEMMI was available at baseline for 356/391 (91.1%), at discharge for 321/391 (82.1%), at one-month post-discharge for 278/391 (71.1%) and at three months post-discharge for 226/391 (57.8%) participants. At three months post-discharge 37 (9.0%) participants were deceased and 189 participants (48.3%) were lost to follow up.

For the 391 participants (men: n = 201; 51.4%, women: n = 190; 48.6%) the mean (sd) age was 79.7 (6.7) years. The median (IQR) length of stay was 5.7 (3.9–8.9) days. At baseline, DEMMI (mean (sd)) score was 55.8 (23.0) points for all participants with a significant difference between men and women (mean (SD) men 58.2 (23.2) points, women 53.3 (22.3) points; p = 0.04) (Table 1).

**Table 1:** Baseline characteristics of the study population.

	All participants	Men	Women
	(n = 391)	(n = 201)	(n = 190)
Age (years), mean (SD)	79.6 (6.7)	79.2 (6.4)	80.1 (6.9)
Living arrangements before admission N (%)			
Independent	332 (84.9)	181 (90.0)	201 (79.5)
Nursing home	8 (2.0)	2 (1.0)	6 (3.2)
Senior residence/Assisted living	51 (13.0)	18 (9.0)	33 (17.4)
Marital status N (%)			
Married or living together	205 (52.4)	142 (70.6)	63 (33.2)
Single or divorced	60 (15.3)	22 (10.9)	38 (20.0)
Widow/widower	126 (32.2)	37 (18.4)	89 (46.8)
Primary admission diagnosis, N (%)			
Cardiovascular disease	121 (30.9)	66 (32.8)	55 (28.9)
Gastrointestinal disease	43 (11.0)	21 (10.4)	22 (11.6)
Pulmonary disease	71 (18.2)	34 (16.9)	37 (19.5)
Infection	56 (14.3)	30 (14.9)	26 (13.7)
Other	100 (25. 6)	50 (24.9)	50 (26.3)
Education N (%)			
Primary school	99 (25.3)	42 (20.9)	57 (30.0)
Elementary technical/domestic science school	86 (22.0)	46 (22.9)	40 (21.1)
Secondary vocational education	116 (29.7)	55 (27.4)	61 (32.1)
Higher level high school/third level education	90 (23.0)	58 (28.9)	32 (16.8)
Body Mass Index (kg/m^2^), mean (SD)	25.2 (5.1)	25.0 (4.9)	25.5 (5.2)
Length of stay (days), median (IQR)	5.7 (3.9–8.9)	5.8 (3.8–8.1)	5.7 (3.9–10.1)
Charlson comborbidity index (CCI), mean (SD)	2.2 (2.0)	2.3 (2.0)	2.1 (1.9)
Nutrition (SNAQ), mean (SD)	1.6 (1.8)	1.5 (1.8)	1.7 (1.8)
Mobility (DEMMI) (n = 356), mean (SD)	55.8 (23.0)	58.2 (23.8)	53.3 (22.3)*
Mobility (DEMMI) (n = 356), median (IQR)	57 (41–74)	62 (41–74)	57 (40–67)
Grip strength (JAMAR in kg) (n = 368), mean (SD)	27.3 (10.8)	33.9 (10.1)	20.2 (5.9)*
MMSE cognitive impairment, mean (SD)	25.9 (3.2)	26.2 (3.2)	25.6 (3.3)
Depressive symptoms (GDS), mean (SD)	4.0 (2.9)	3.5 (2.7)	4.4 (3.0)*
Fatigue (NRS), mean (SD)	5.4 (2.9)	4.9 (2.9)	5.9 (2.7)*
Fear of Falling (NRS), mean (SD)	3.0 (3.3)	2.2 (3.1)	3.7 (3.4)*
KATZ 6 ADL, median (IQR)	1 (0–3)	0.5 (0–2)	1 (0–3)*

Abbreviations: SD = Standard Deviation; IQR = Interquartile range; Body Mass Index (BMI) = weight / square of the body height in kg/m2; CCI = Charlson comorbidity index range 0–31 with a higher score indicating more comorbidity; SNAQ = Short Nutritional Assessment Questionnaire range 0–5; DEMMI = De Morton Mobility Index range 0–100 with a higher score indicating better mobility; MMSE = Mini Mental State Examination range 0–30 with a higher score indicating less cognitive impairment; GDS = Geriatric Depression Scale range 0–15 with a higher score indicating more depressive symptoms; Fatigue NRS = Numeric Rating Scale range 0–10. Fear of Falling NRS = Numeric Rating Scale range 0–10 with higher score on the NRS indicating more fatigue or fear of falling. KATZ 6 ADL = Activities of Daily Living range 0–6 with a higher score indicating more disabilities.

* p-value<0.05; Independent T-test and Mann-Whitney U test were used for continues and categorical variables.

### Course of mobility

**Fig 2:**
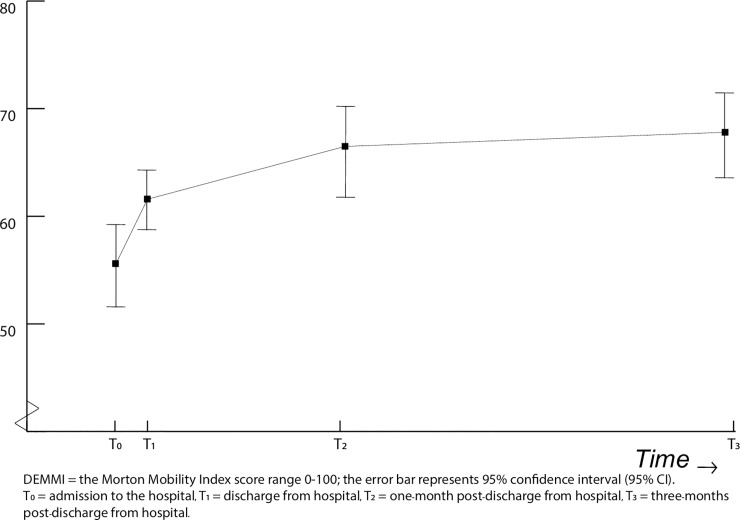
Course of mobility from admission up to three months post-discharge.

Linear Mixed Model showed a significant improvement in the course of mobility after hospital admission up to three months post-discharge; with a progression in DEMMI score of 57 to 62 points from admission to discharge, towards a score of 67 points at one-month and 68 points at three months post-discharge (Fig 2). At three months post-discharge, 74 out of 226 (40.1%) participants scored lower than 74 points on the DEMMI, indicating a mobility level below the normative level for independent living [21].

### Association between muscle strength and mobility

Table 2 shows that in the crude model, a longitudinal association between muscle strength and course of mobility up to three months post-discharge was found (beta = 0.64; p<0.01). This means that a difference of one-kilogram in muscle strength is associated with a difference of 0.64 points on the DEMMI. There were no significant differences of the beta in the association between muscle strength and mobility at different time-points.

**Table 2:** Longitudinal association of muscle strength with course of mobility.

		All participants (n = 391)	Men (n = 201)	Women (n = 190)
Model 1: crude model	Parameter	beta* (95% CI)	beta* (95% CI)	beta* (95% CI)
	Grip strength	0.64 (0.50–0.79)	0.55 (0.35–0.76)	1.19 (0.85–1.53)
**Model 2 (adjusted): influence**	Age	0.51** (0.36–0.66)	0.37** (0.15–0.58)	1.02** (0.67–1.37)
**per factor on grip strength**	Marital Status	0.59 (0.44–0.74)	0.55 (0.35–0.75)	1.14 (0.80–1.49)
	Living Arrangement	0.60 (0.50–0.79)	0.53 (0.33–0.74)	1.13 (0.79–1.48)
	Educational level	0.63 (0.48–0.78)	0.55 (0.35–0.75)	1.17 (0.83–1.52)
	Body Mass Index	0.69 (0.54–0.85)	0.57 (0.36–0.78)	1.27 (0.91–1.63)
	Comorbidity	0.63 (0.48–0.78)	0.53 (0.32–0.73)	1.15 (0.81–1.49)
	Cognitive impairment	0.53** (0.39–0.68)	0.45** (0.25–0.66)	0.98** (0.64–1.32)
	Depressive symptoms	0.56** (0.42–0.71)	0.51 (0.31–0.72)	0.97** (0.63–1.30)
	Fear of Falling	0.55** (0.41–0.70)	0.50 (0.30–0.70)	1.08** (0.75–1.42)
	Fatigue	0.60 (0.45–0.74)	0.56 (0.37–0.76)	1.05** (0.71–1.38)
	Nutrition	0.62 (0.48–0.77)	0.56 (0.35–0.76)	1.13 (0.78–1.48)
	Length of Stay	0.62 (0.46–0.56)	0.52 (0.30–0.74)	1.18 (0.84–1.52)
**Model 3: final model with**	Grip strength	0.35** (0.20–0.49)	0.32** (0.10–0.54)	0.68** (0.35–1.01)
**confounders**		(age, cognitive impairment, depressive symptoms, fear of falling)	(age, cognitive impairment)	(age, cognitive impairment, depressive symptoms, fear fear of falling, fatigue)

CI = Confidence interval.

* P-value below <0.01.

** Beta more than 10% different from beta in crude model

Gender was determined as effect modifier (muscle strength*Gender, beta = 0.73; p<0.01) and therefore, the analysis for men and women are presented separately. The crude model of the association showed different associations for men (beta = 0.55; p<0.01) and women (beta = 1.19; p<0.01) respectively. Age and cognitive impairment were identified as confounders for both men and women. For women only, also depressive symptoms, fear of falling and fatigue were identified as confounders. Marital status, living arrangement, educational level, body mass index, comorbidity, nutrition and length of stay did not influence the beta in the association of muscle strength and mobility.

### Sensitivity analysis

At baseline, 52 out of 391 (13.3%) participants had decreased grip strength. For participants with low muscle strength at baseline, the association between muscle strength and course of mobility did not change substantially.

## Discussion

This multicentre cohort study yielded three clinical important findings. First, the level of mobility improved significantly in acute hospitalized older adults from admission up to three months post-discharge. Second, muscle strength was longitudinally associated with the course of mobility up to three months post-discharge. Third, the association between muscle strength and the course of mobility was different in men and women, confounded by age and cognitive impairment for both women and men whereas for women, also, fear of falling and depressive symptoms confounded the association. These findings highlight that multiple factors play a role in regaining mobility after acute hospitalization.

During hospitalization, the observed improvement of the level of mobility, was in line with two other studies [6,8]. After hospitalization, however, the course of mobility differed. In contrast with our study, Bodilsen et al. [6] found that mobility stabilized up to one-month post-discharge. An explanation for the difference could be that they used the Timed Up and Go test as measurement tool, which focuses on standing up from a chair and walking instead of a broader spectrum of mobility such as transfers out of bed, balance tests and walking for a longer time. Moen et al. [8] reported mobility only at two time-points: baseline and three weeks post-hospital. Although several studies reported regaining pre-admission mobility can take up to three months, there is currently no study reporting in detail on the course of mobility up to three months post-discharge [3]. Our study provides novel information that the largest improvement occurs during hospitalization and in the first month post-discharge and stabilises up to three months post-discharge.

Muscle strength was found to be associated with the course of mobility up to three months. This finding is in accordance with a previous study where muscle strength is considered as ‘vital sign’ of poor performance and is associated with reduced mobility [9]. Our study adds to this that the association between muscle strength and course of mobility is consistent during the first three months post-discharge and substantially influenced by several factors. It was reported previously [4] that several factors may affect the mobility after hospitalization but the interaction between the factors was not described until now.

Our study is consistent with the hypothesis [6,29] that muscle strength is an important target for interventions. It has been shown that interventions that focus on increasing muscle strength, particularly progressive resistance training may be beneficial to restore mobility, even in vulnerable older adults [29]. However, our study showed that besides muscle strength, factors such as age, cognition, depressive symptoms, fear of falling and fatigue should be taken into account in the development and application of exercise intervention. Depressive symptoms, fear of falling and fatigue may be barriers to start exercises and regain mobility after hospitalization.

### Strength and limitations of the study

The key strength of this study is the multicenter longitudinal design, with multiple measurements up to three months post-discharge. It needs to be acknowledged that the study has several limitations. Firstly, information was lacking regarding mobility prior to admission, hence it was not possible to compare mobility post-discharge with pre-admission levels. Secondly, data was not available for all older adults at all time points. This is a well-known challenge in research in geriatric population and is difficult to avoid [30]. Data was missing because of death, refusal or deterioration in health and could have influenced our results. However, application of advanced statistical analysis has the advantage of its ability to deal with missing data and provides unbiased results. Thirdly, our selection criteria may have an effect on the generalizability of the study. Participants with a score of 15 or lower on the MMSE scale were excluded. As a consequence, the most vulnerable older adults may have been excluded. Fourthly, no data was available after three months post-discharge so it is unknown if the association continues over a longer time.

## Conclusion

Muscle strength is longitudinally associated with the course of mobility even after controlling for factors as cognitive impairment, depressive symptoms, fatigue and fear of falling. Interventions to improve mobility including muscle strength are warranted, in acute hospitalized older adults.
